# Risky sexual behavior and associated factors in undergraduate students in a city in Southern Brazil

**DOI:** 10.11606/s1518-8787.2020054001709

**Published:** 2020-04-03

**Authors:** Débora Dalmas Gräf, Marilia Arndt Mesenburg, Anaclaudia Gastal Fassa

**Affiliations:** I Universidade Federal de Pelotas Programa de Pós-Graduação em Epidemiologia PelotasRS Brasil Universidade Federal de Pelotas. Programa de Pós-Graduação em Epidemiologia. Pelotas, RS, Brasil; II Universidade Federal de Ciências da Saúde de Porto Alegre Porto AlegreRS Brasil Universidade Federal de Ciências da Saúde de Porto Alegre. Porto Alegre, RS, Brasil; III Universidade Federal de Pelotas Faculdade de Medicina Departamento de Medicina Social PelotasRS Brasil Universidade Federal de Pelotas. Faculdade de Medicina. Departamento de Medicina Social. Pelotas, RS, Brasil

**Keywords:** Student Health, Universities, Health Risk Behaviors, Unsafe Sex, Sexually Transmitted Infections

## Abstract

**OBJECTIVE:**

To describe the sexual behavior of freshmen undergraduate students according to demographic, economic, psychosocial and behavioral characteristics, and evaluate the prevalence of risky sexual behavior and its associated factors.

**METHODS:**

A cross-sectional study of the census type with undergraduate students over 18 years old of 80 undergraduate courses of the Universidade Federal de Pelotas (UFPel), in Rio Grande do Sul (RS), who entered in the first semester of 2017 and remained enrolled in the second semester. Undergraduate students who reported having had sex were evaluated. We considered as risky sexual behavior having more than one sexual partner within the last three months and not having used condoms in the last sexual intercourse.

**RESULTS:**

The prevalence of risky sexual behavior was 9% (95%CI 7.6–10.5). Men presented more risky behavior than women, with a prevalence of 10.8% and 7.5%, respectively. Of the undergraduate students, 45% did not use condoms in the last sexual intercourse, and 24% had two partners or more within three months before the survey. Smartphone applications for sexual purposes were used by 23% of students within three months before the survey. Risky sexual behavior was associated with gender, age at first sexual intercourse, frequency of alcohol consumption, consumption of psychoactive substances before the last sexual intercourse and use of smartphone applications for sexual purposes.

**CONCLUSION:**

Although undergraduate students are expected to be an informed population, the prevalence of risky sexual behavior was important, indicating the need to expand public investment in sexual education and awareness actions.

## INTRODUCTION

Risky sexual behaviors (RSB), such as unsafe sexual intercourse and multiplicity of partners are more frequent among adolescents and young adults (between 15 and 24 years old). Factors associated with the admission to the university may increase the occurrence of RSB, since they imply a series of social changes in the individual’s life^1^ .

RSB may result in sexually transmitted infections (STIs)^2 , 3^ and unplanned pregnancy. STIs are among the most prevalent acute conditions in the world, with about one million new cases per day^4^ . Brazil has been experiencing a resurgence of STIs, especially human immunodeficiency virus (HIV) and syphilis, with a significant increase among young people aged between 15 and 29 years old^5 , 6^ . The main consequences of STIs are infertility, ectopic pregnancy, stillbirths, pelvic inflammatory disease and neurological and cardiovascular implications in adults^4^ . On the other hand, unplanned pregnancy is particularly problematic in younger age groups, as it compromises the completion of school and academic life, besides increasing the risk of complications in the pregnancy itself^7^ .

In Brazil, the evaluation of a representative sample of high school students indicated that 32% of the students did not use condoms in sexual intercourses that occurred in the month before the survey^8^ . Among undergraduate students, non-use of condom in the last sexual intercourse ranged from 85.7% to 38.6%^2 , 9^ . The frequency of students who had between one and three sexual partners within three months before the survey was 95% in women and 89% in men^10^ . In the United States, 48% of undergraduate students used condoms in the last sexual intercourse, and the prevalence of risky sexual behavior was 14%, considering those who reported not using condoms in the last intercourse and having had more than one partner within the last 12 months prior to the survey^11^ .

A study conducted in 31 U.S. higher education institutions indicated that 44% of students had more than one partner within three months before the survey, and 16% used psychoactive substances (alcohol or illicit drugs) before the last sexual intercourse^12^ . The use of psychoactive substances among Brazilian undergraduate students before the last intercourse was similar, around 15%^2^ . In the municipality of Pelotas, a survey with adolescents between 15 and 18 years old indicated that 10.7% ingested alcoholic beverages before the last intercourse and only 56% of adolescents used condoms in the last three sexual relationships^13^ .

The non-use of condoms in both high school and undergraduate students was positively associated with males, alcohol intake and multiplicity of partners^1 , 14^ and inversely associated with the age of the individual and with socioeconomic level^15 , 16^ .

Studies evaluating risky sexual behavior in Brazil are predominantly among school adolescents. Those who evaluated undergraduate students had a descriptive approach and their sample was mostly composed of students from the health area or by convenience, focusing on the evaluation of other outcomes related to sexual behavior such as the level of knowledge regarding STIs^9 , 16^ . Moreover, the impact of the psychosocial characteristics of undergraduate students, such as sexual orientation and gender identity, the variability between the areas of knowledge and the role of technology on sexual behaviors, is little addressed.

Our study seeks to identify the main characteristics of the sexual behavior of freshmen undergraduate students according to gender and verify the prevalence of risky sexual behavior, as well as the main sociodemographic and behavioral associated factors, in a census of freshmen students of the Universidade Federal de Pelotas (UFPel), in Southern Brazil.

## METHODOLOGY

A cross-sectional, census-type study was conducted with students entering higher education in face-to-face courses of the Universidade Federal de Pelotas (UFPel), offered in the first semester of 2017. In total, 3,424 vacancies were offered in 83 undergraduate courses. The census was conducted in the context of the research consortium of the Graduate Program in Epidemiology of UFPel, a method of data collection of a single population for the development of the study of a class of masters’ students.

Students who entered UFPel in the first semester of 2017, were regularly enrolled in a face-to-face undergraduate course in the second semester of 2017 and who understood Portuguese were included in our study. Students under the age of 18 and those who had classes taught outside the city of Pelotas or Capão do Leão were excluded.

Data collection was anonymously performed during class hours through a self-completed questionnaire on tablets using the Research Electronic Data Capture (REDCap) tool. The research instrument included demographic, economic, psychosocial, academic and behavioral issues, as well as the description of the students’ sexual behavior.

A questionnaire with ten objective questions to characterize sexual behavior was prepared based on the Global School-Based Student Health Survey (GSHS) and the Youth Risk Behavior Survey (YRBS) and applied only to students who had already started their sex life. This questionnaire evaluated the age at first sexual intercourse, number of partners within the three months before the survey, condom use in the last intercourse, practice of anal sex in the last intercourse, use of alcohol or illicit drugs in the last intercourse, use of contraceptive method, HIV testing at some point in life, reason for testing, diagnosis of STIs at some point in life, and use of smartphone applications with the purpose of having sex within the three months prior to the survey.

We considered as risky sexual behavior to have more than one sexual partner within three months before the survey combined with the non-use of condoms (male or female condoms) in the last sexual intercourse. This operationalization of the outcome sought to restrict the evaluation to objective aspects related to the risk to the individuals’ health.

The exposure variables were biological sex (female, male), age (divided into the following age groups: 18 to 19 years, 20 to 22 years, 23 to 25 years, 26 years or more), skin color (white, black, brown/other), sexual orientation (heterosexual/asexual, homosexual, bisexual), gender identity (cisgender, transgender, non-binary gender), relationship status (not in any relationship, casual dating, in a relationship, living with spouse or boyfriend/girlfriend), economic class (classification of the *Associação Brasileira das Empresas de Pesquisa – ABEP* ), type of high school (public, private), where the student lived in the year before entering UFPel (Pelotas, another city in Rio Grande do Sul, another state of Brazil/another country), if the student follows some religious doctrine (no, yes), who the student lives with (family, friends/colleagues, alone), tobacco consumption (never smoked, smoker, former smoker), frequency of alcohol consumption (never, once a month or less, two to four times a month, two to three times a week, four or more times a week) and area of knowledge to which the course attended belongs (exact and earth/agrarian sciences, health and biological sciences, applied social sciences and humanities, linguistics, languages and literature and arts).

At first, descriptive analysis of the demographic, economic, psychosocial, academic and behavioral characteristics of the students was performed using absolute and relative frequency. The chi-square test for heterogeneity was used to evaluate the differences between the sexes.

Multivariate analysis using Poisson regression with robust variance was performed to evaluate factors associated with the occurrence of risky sexual behavior, since this is the best alternative for the analysis of cross-sectional studies with dichotomous outcomes and provides a good estimate of the prevalence ratio^19^ . The analysis followed a previously defined conceptual model, with stepwise backward selection. Demographic, economic and psychosocial characteristics were included in the first level; variables of the academic context and age at first sexual intercourse in the second level; and behavioral aspects in the third level. The variables associated with the outcome with p-value < 0.20 were maintained in the multivariable model for control of confounding factors. Associations with p-value < 0.05 were considered significant. Data analysis was performed in the statistical program Stata Statistical Software, version 14.0.

Our study is in accordance with resolution 466/2012 of the National Health Council and was approved by the Research Ethics Committee of the Faculdade de Medicina da UFPel, opinion number 2,352,451.

## RESULTS

In the first semester of 2017, 3,212 students entered UFPel undergraduate courses. Of these, 2,706 remained enrolled at the beginning of our study, which occurred between November 2017 and July 2018. At the end of the census of the new students, 1,865 students were interviewed, corresponding to a 69% response rate. Of the remaining 31%, only 1.8% refused to participate. The others were students who were not found during the field work period after several attempts. Approximately 47% of the losses were women, 24% were between 18 and 19 years old, 29% were between 20 and 22 years old and 46.7% were 23 years or older. Exact and earth/agrarian sciences was the area of knowledge with the highest number of losses, with 38.3%, followed by the applied and human social sciences, with 38.4%, linguistics, languages and literature and arts, with 16%, and health and biological sciences, with 11%. The high rates of absenteeism and dropout rates from the first to the second semester of school, often without formalization, limited the response rate.

Among the survey respondents, 1,547 students (83.5% of respondents) said they had already had sex. Of these, 72.2% were white, 37.5% were between 18 and 19 years old, 44.3% were in economy class B, 36.4% were dating, and 13.4% were married or shared housing with their spouse. The sexual orientation of minorities (homosexual or bisexual) was reported by 22.2%, and 10.4% did not identify with the gender which was attributed at birth (transgender or non-binary). Regarding the area of knowledge, 28.3% were inserted in courses of the exact and earth/agrarian sciences, 18% of the health and biological sciences, 35.2% of the applied social sciences and humanities and 18.5% of linguistics, languages and literature and arts, as shown in Table 1 . Of the students, 37% had their first sexual relationship before the age of 15. Regarding the use of substances during the last sexual intercourse, 15% used alcoholic beverages, 2.7% illicit drugs, and 5.8% used both. About 12% of the undergraduate students had anal sex during their last sexual intercourse, and 22% used smartphone applications to seek for sex within three months before the survey. According to the interviewees, 23% had two or more sexual partners within three months before the survey, and 45% did not use condoms in the last sexual intercourse, as shown in Table 2 .

**Table 1:** Characterization of freshmen undergraduate students according to biological sex (N = 1,547), Universidade Federal de Pelotas, 2017–2018.

Characteristics	Total	Male	Female	P-value^a^
		
	N (%)	N (%)	N (%)	
Age				
18 to 19 years	577 (37.5)	252 (35.6)	325 (39.0)	0.293
20 to 22 years	509 (33.1)	228 (32.4)	280 (33.6)	
23 to 25 years	189 (12.3)	95 (13.5)	94 (11.3)	
26 years	263 (17.1)	128 (18.2)	134 (16.1)	
Skin color				
White	1,116 (72.2)	508 (71.8)	606 (72.6)	0.507
Black	201 (13.0)	88 (12.4)	113 (13.5)	
Mixed or other	228 (14.8)	112 (15.8)	116 (13.9)	
Relationship status				
Not in any relationship	509 (33.0)	282 (39.8)	227 (27.3)	<0.001
Casual dating	266 (17.2)	130 (18.4)	136 (16.3)	
In a relationship	562 (36.4)	212 (29.9)	350 (42.0)	
Living with spouse or boyfriend/girlfriend	206 (13.4)	84 (11.9)	120 (14.4)	
Sexual orientation				
Heterosexual or asexual	1,198 (77.8)	575 (81.2)	622 (74.9)	< 0.001
Homosexual	129 (8.4)	86 (12.2)	43 (5.2)	
Bisexual	212 (13.8)	47 (6.6)	165 (19.9)	
Gender identity				
Cisgender	1,382 (89.6)	626 (88.4)	756 (90.5)	0.385
Transgender	110 (7.1)	56 (7.9)	54 (6.5)	
Non-binary gender	51 (3.3)	26 (3.7)	25 (3.0)	
Economic class^b^				
A	221 (15.0)	115 (17.1)	106 (13.2)	0.062
B	653 (44.3)	303 (45.1)	349 (43.6)	
C	536 (36.3)	223 (33.2)	312 (39.0)	
D and E	65 (4.4)	31 (4.6)	34 (4.2)	
Type of high school				
Public	1,136 (73.5)	510 (72.0)	624 (74.6)	0.248
Private	410 (26.5)	198 (28.0)	212 (25.4)	
Place of residence before entering UFPel				
Pelotas	704 (45.6)	314 (44.3)	388 (46.5)	0.128
Another city of RS	536 (34.7)	239 (33.7)	297 (35.6)	
Another state of Brazil or another country^c^	305 (19.7)	156 (22.0)	149 (17.9)	
Who they live with				
Family members (parents, siblings or spouses)	938 (60.8)	411 (58.0)	525 (63.0)	0.092
Friends or colleagues	414 (26.8)	199 (28.1)	215 (25.8)	
Alone	192 (12.4)	99 (14.0)	93 (11.2)	
Religion				
No	1,086 (70.3)	540 (76.2)	546 (65.4)	< 0.001
Yes	460 (29.7)	169 (23.8)	289 (34.6)	
Tobacco Consumption				
Never smoked	1,073 (69.4)	469 (66.2)	603 (72.1)	0.002
Smoker	194 (12.6)	111 (15.7)	83 (9.9)	
Former smoker	279 (18.0)	129 (19.2)	150 (17.9)	
Consumption of alcoholic beverages^d^				
Never	126 (8.6)	62 (9.0)	64 (8.3)	0.055
Once a month or less	388 (26.5)	162 (23.4)	226 (29.2)	
Two to four times a month	585 (39.9)	275 (39.8)	309 (39.9)	
Two to three times a week	296 (20.2)	152 (22.0)	144 (18.6)	
Four or more times a week	71 (4.8)	40 (5.8)	31 (4.0)	
Area of knowledge				
Exact and earth/agrarian sciences	437 (28.3)	238 (33.6)	198 (23.7)	< 0.001
Health and biological sciences	279 (18.0)	115 (16.2)	164 (19.6)	
Applied social sciences and humanities	545 (35.2)	227 (32.0)	318 (38.0)	
Linguistics, language and literature and arts	286 (18.5)	129 (18.2)	156 (18.7)	

^a^ Chi-square test for heterogeneity for differences between the sexes

^b^ According to the classification of the *Associação Brasileira das Empresas de Pesquisa (ABEP)*

^c^ The number of students who lived in another country was n = 3

^d^ The maximum number of missings was n = 81 for this variable

**Table 2:** Description of sexual behavior in freshmen undergraduate students according to biological sex (N = 1,547), Universidade Federal de Pelotas, 2017–2018.

Outcomes	Total	Male	Female	P-value^a^
		
	N (%)	N (%)	N (%)	
Sex life initiation^b^				
No	305 (16.5)	124 (14.9)	181 (17.8)	0.102
Yes	1,547 (83.5)	709 (85.1)	836 (82.2)	
Age at first sexual intercourse				
13 years or less	82 (5.3)	54 (7.7)	28 (3.4)	< 0.001
14 to 15 years	483 (31.3)	239 (33.9)	244 (29.3)	
16 to 17 years	583 (37.8)	264 (37.5)	318 (38.1)	
18 to 19 years	295 (19.1)	113 (16.0)	181 (21.7)	
20 years or more	98 (6.4)	35 (5.0)	63 (7.6)	
Use of substances^c^				
No	1,178 (76.4)	522 (73.9)	654 (78.5)	0.011
Alcoholic beverages	231 (15.0)	107 (15.2)	124 (14.9)	
Illicit drugs	42 (2.7)	21 (3.0)	21 (2.5)	
Alcoholic beverages and illicit drugs	90 (5.8)	56 (7.9)	34 (4.1)	
Anal sex practice^c,d^				
No	1,354 (87.9)	571 (81.1)	781 (93.7)	< 0.001
Yes	186 (12.1)	133 (18.9)	53 (6.3)	
Use of contraceptive method^c^				
No	574 (37.3)	291 (41.3)	283 (33.9)	< 0.001
Oral contraceptive pills	728 (47.2)	293 (41.6)	433 (51.9)	
Coitus interruptus	62 (4.0)	21 (3.0)	41 (4.9)	
Morning-after pill	48 (3.1)	21 (3.0)	27 (3.2)	
Injectable contraceptive	31 (2.0)	11 (1.6)	20 (2.4)	
Intrauterine device	15 (1.0)	10 (1.4)	5 (0.6)	
Calendar method	12 (0.8)	2 (0.3)	10 (1.2)	
Other	26 (1.7)	15 (2.1)	11 (1.3)	
Does not know	45 (2.9)	41 (5.8)	4 (0.5)	
Use of modern method^c,e^				
No	719 (46.7)	370 (52.5)	349 (41.9)	< 0.001
Yes	822 (53.3)	335 (47.5)	485 (58.1)	
Use of smartphone applications^f^				
No	1,193 (77.3)	453 (64.2)	738 (88.4)	< 0.001
Yes	350 (22.7)	253 (35.8)	97 (11.6)	
Number of partners^d,f^				
None	218 (14.2)	105 (14.9)	113 (13.6)	< 0.001
Only 1 partner	950 (61.7)	382 (54.1)	566 (68.0)	
2 to 3 partners	245 (15.9)	130 (18.4)	115 (13.8)	
4 or more partners	127 (8.3)	89 (12.6)	38 (4.6)	
Condom^c^				
No	700 (45.4)	280 (39.7)	418 (50.1)	< 0.001
Yes	842 (54.6)	426 (60.3)	416 (49.9)	
Risky sexual behavior^g^				
No	1,402 (91.0)	630 (89.2)	770 (92.5)	0.025
Yes	138 (9.0)	76 (10.8)	62 (7.5)	

^a^ Chi-square test for heterogeneity for differences between the sexes

^b^ Number of observations: 1,862.

^c^ In the last sexual intercourse

^c^ The maximum number of missings was n = 20 for this variable

^e^ Considered as pill, intrauterine device, injectable contraceptive and morning-after pill

^f^ Within three months prior to the survey

^g^ More than one partner within three months before the survey and have not used condoms in the last sexual intercourse

Among male students, 41.6% had their first sexual intercourse before the age of 15, 31% had two or more sexual partners within three months before the survey and about 40% reported having not used condoms in the last sexual intercourse. Of the interviewees, 20% had anal sex in the last intercourse and 35.8% used smartphone applications to seek for sex within three months prior to the survey, also shown in Table 2 .

Among female students, 33% had their first sexual intercourse before the age of 15, 18.4% had two or more sexual partners within three months before the survey and 50% did not use condoms in the last sexual intercourse. Of the undergraduate students, 6% had anal sex in the last intercourse and 11.6% used smartphone applications to seek for sex.

The prevalence of risky sexual behavior among freshmen undergraduate students was 9% (95% confidence interval [95%CI] 7.6–10.5), 10.8% among male students, and 7.5% among female students, also shown in Table 2 .

In the multivariate analysis, men had 48% (Odds Ratio [RO] 1.48; 95%CI 1.07–2.07) more risky sexual behavior than women. The age at which students started sex life was inversely associated with RSB. The frequency of alcohol consumption was directly associated with risky sexual behavior, and those who consumed alcohol four or more times a week had a five-fold higher chance of having RSB (RO 5.10; 95%CI 1.49–17.6). The use of illicit drugs in the last sexual intercourse and the use of smartphone applications increased by more than 100% the chance of presenting RSB (RO 2.23; 95%CI 1.14–4.39 and RO 2.57; 95%CI 1.76–3.74, respectively). Age, sexual orientation, where the student lived before entering UFPel, tobacco use and anal sex practice in the last sexual intercourse were not associated with RSB in the adjusted analysis, as shown in Table 3 .

**Table 3:** Description of sexual behavior in freshmen undergraduate students according to biological sex (N = 1,381), Universidade Federal de Pelotas, 2017–2018.

Characteristics	Prevalence of RSB (%)	RSB (crude)			RSB (adjusted)		
		
	%	PR	95%CI	P-value^a^	PR	95%CI	P-value^a^
Level 1							

Sex							
Female	7.5	1	-	0.024	1	-	0.020
Male	10.8	1.44	1.05–1.99		1.48	1.07–2.07	
Skin color							
White	8.3	1	-	0.200	1	-	0.253
Black	9.5	1.15	0.72–1.84		1.14	0.69–1.88	
Mixed or other	11.4	1.44	0.96–2.16		1.42	0.94–2.15	
Gender identity							
Cisgender	8.7	1	-	0.021	1	-	0.051
Transgender	8.2	0.95	0.49–1.81		1.04	0.54–2.00	
Non-binary gender	19.6	2.27	1.27–4.06		2.12	1.16–3.87	
Religion							
No	9.7	1	-	0.127	1	-	0.119
Yes	7.2	0.75	0.51–1.09		0.73	0.49–1.08	
Economic class^b^							
A	11.0	1	-	0.114^c^	1	-	0.091^c^
B	9.4	0.86	0.55–1.34		0.88	0.56–1.38	
C	7.5	0.68	0.42–1.11		0.68	0.42–1.11	
D and E	7.7	0.70	0.28–1.77		0.66	0.26–2.68	

Level 2							

Who they live with							
Family members (parents, siblings or spouses)	7.5	1	-	0.031	1	-	0.137
Friends or colleagues	11.9	1.59	1.12–2.24		1.42	0.99–2.05	
Alone	9.9	1.32	0.81–2.14		1.34	0.83–2.17	
Area of knowledge							
Exact and earth/agrarian sciences	7.3	1	-	0.355	1	-	0.144
Health and biological sciences	9.0	1.23	0.74–2.02		1.18	0.70–1.99	
Applied social sciences and humanities	9.0	1.23	0.80–1.89		1.32	0.85–2.03	
Linguistics, language and literature and arts	11.3	1.54	0.96–2.45		1.75	1.08–2.84	
Age at first sexual intercourse							
13 years or less	17.1	1	-	< 0.001	1	-	0.001
14 to 15 years	12.2	0.72	0.42–1.22		0.80	0.46–1.39	
16 to 17 years	7.9	0.46	0.26–0.80		0.51	0.28–0.90	
18 to 19 years	4.8	0.28	0.14–0.56		0.28	0.13–0.60	
20 years or more	5.1	0.30	0.11–0.79		0.34	0.13–0.90	

Level 3							

Consumption of alcoholic beverages							
Never	2.4	1	-	< 0.001^c^	1	-	< 0.001^c^
Once a month or less	5.9	2.49	0.76–8.16		2.30	0.69–7.66	
Two to four times a month	6.9	2.90	0.91–9.23		2.34	0.72–7.65	
Two to three times a week	17.2	7.23	2.30–22.7		4.30	1.34–13.8	
Four or more times a week	25.4	10.65	3.25–34.9		5.10	1.49–17.6	
Substance use in the last intercourse							
No	6.1	1	-	< 0.001	1		0.024
Alcoholic beverages	16.1	2.63	1.81–3.80		1.56	1.06–2.33	
Illicit drugs	19.1	3.11	1.60–6.03		2.23	1.14–4.39	
Alcoholic beverages and illicit drugs	23.3	3.81	2.46–5.89		1.49	0.91–2.47	
Use of smartphone applications within the last three months							
No	5.6	1	-	< 0.001	1	-	< 0.001
Sim	20.6	3.71	2.71–5.06		2.57	1.76< –3.74	

RSB: risky sexual behavior (more than one partner within three months before the survey and having not used condoms in the last sexual intercourse); PR: prevalence ratio

^a^ Chi-square test for heterogeneity

^b^ According to the classification of the *Associação Brasileira das Empresas de Pesquisa (ABEP)*

^a^ Chi-square test for heterogeneity

Approximately 9% of undergraduate students have had at least one sexually transmitted infection at some point in life. Among the students who had had STIs, 34.8% reported HPV, 15.6% genital herpes and 13.3% gonorrhea, with herpes being more prevalent in females and gonorrhea in males, as shown in Table 4 . Among undergraduate students, 38% reported having already undergone HIV testing at some time in their life. The most common reasons for taking the test were unprotected sexual intercourse (26%), blood donation and medical request (both with 15.7%), in addition to government actions (13%).

**Table 4:** Frequency of sexually transmitted infections and HIV testing among freshmen undergraduate students, Universidade Federal de Pelotas, 2017–2018.

Outcomes	Total	Male	Female	P-value^a^
		
	N (%)	N (%)	N (%)	
Sexually transmitted infection (N = 1,540)				
No	1,405 (91.2)	643 (91.3)	761 (91.2)	1.000
Yes	135 (8.8)	61 (8.7)	73 (8.8)	
Sexually transmitted infection (N = 135)				
HPV	47 (34.8)	18 (29.5)	28 (38.4)	0.004
Genital herpes	21 (15.6)	6 (9.8)	15 (20.6)	
Gonorrhea	18 (13.3)	14 (23.0)	4 (5.5)	
Chlamydia	7 (5.2)	3 (4.9)	4 (5.5)	
Tricomandiasis	7 (5.2)	1 (1.6)	6 (8.2)	
Syphilis	6 (4.4)	4 (6.6)	2 (2.7)	
HIV/AIDS	5 (3.7)	5 (8.2)	-	
Other	24 (17.8)	10 (1.4)	14 (1.7)	
HIV testing (N = 1,542)^b^				
No	961 (62.3)	444 (63.0)	517 (61.9)	0.673
Yes	581 (37.7)	261 (37.0)	318 (38.1)	
Main reason for HIV testing (N = 580)				
Unprotected sexual intercourse	152 (26.2)	82 (31.4)	70 (22.1)	< 0.001
Blood donation	91 (15.7)	48 (18.4)	43 (13.6)	
Medical request	91 (15.7)	29 (11.1)	62 (19.6)	
Governmental actions	76 (13.1)	37 (14.2)	39 (12.3)	
Prenatal	43 (7.4)	1 (0.4)	42 (13.2)	
Partner request	13 (2.2)	9 (3.5)	4 (1.2)	
Occupational exposure	11 (1.9)	3 (1.1)	7 (2.2)	
Other	103 (17.8)	52 (19.9)	50 (15.8)	

^a^ Chi-square test for heterogeneity for differences between the sexes

^b^ The maximum number of missings was n = 20 for this variable; test performed at some point in life

## DISCUSSION

Risky sexual behavior has different definitions in the literature, making comparisons difficult. Moreover, some factors treated as characteristics of sexual behavior are addressed in other studies as part of a risky sexual behavior. The study with the most similar definition to that addressed in our study was conducted with American undergraduate students, considering the same criteria to compose the outcome, but with a period for multiple partners expanded to 12 months. The prevalence of RSB found in this study was 14% and the non-use of condoms in the last sexual intercourse was 52%^11^ , being similar to the findings in the freshmen undergraduate students of UFPel. This prevalence in undergraduate students, a population with a high level of information, reinforce the idea that having information is a necessary factor, but not sufficient to modify behaviors^18^ .

This study agrees with the literature that indicates that half of the undergraduate students used condoms^20 , 21^ and 15% ingested alcoholic beverages in their last sexual intercourse^2 , 12^ . However, a study showed that more than 40% of American college freshmen have had multiple sexual partners within three months before the survey, twice as many as found in our study^12^ . No further studies on the use of smartphone applications to seek for sex were found; however, these resources have been recently made available and, probably, their use is on the rise among undergraduate students. Given this context, the prevalence was considered high.

Risky sexual behavior was positively associated with males, use of psychoactive substances before the last sexual intercourse and use of smartphone applications to seek for sex within three months before the survey. It was directly associated with the frequency of alcohol consumption and inversely associated with the age of sexual debut.

The higher frequency of sexual risk behavior in males agrees with the literature that points to this association regardless of how RSB is evaluated, as well as when examining the components of RSB separately (lower condom use and greater number of partners)^1 , 8 , 11^ . Being male implies being subjected to a repertoire of social pressures, such as encouraging the expression of sexuality in the name of “masculinity,” manhood proof and heterosexuality, resulting in the increase in the number of partners^1 , 22^ . Moreover, some men report that condoms reduce sensitivity during sexual intercourse, contributing to its non-use.

Similar to our study, the literature points to an inverse association between the age at first sexual intercourse and risky sexual behavior. There are indications that not living with a mother or father increases the chances of having an earlier sexual debut. The lower presence and supervision of parents can have a negative impact on children’s sex education^22 , 23^ .

The positive association between the consumption of alcohol and the use of psychoactive substances with RSB agrees with the literature, which points out that these substances potentiate risky behaviors^2 , 10 , 12 , 24^ . This is because narcotic substances, especially alcohol, depress the central nervous system, impairing psychomotor skills and information processing, affecting the perception of danger and the ability to make appropriate decisions^25^ .

Studies on the use of smartphone applications to seek for sex are scarce, as this phenomenon arose approximately 10 years ago, with the popularization of social networks, and migrated from comprehensive platforms to specific communities for this purpose. Studies indicate that applications expand the possibility of intercourse between casual partners and increase the chance of unsafe sex^26 , 27^ , since they facilitate social interactions between unknown individuals. The association between the use of smartphone applications and RSB stands out because despite the high schooling level of this population and the report of having casual sexual partners, they do not use condoms regularly.

All characteristics associated with RSB – male sex, age at first sexual intercourse, high frequency of alcohol consumption, use of psychoactive substances before the last sexual intercourse and the use of smartphone applications to seek for sex – are potential markers of individuals with risk-taking profiles^11^ .

Sexual orientation, gender identity and anal sex practice were not associated with risky sexual behavior. The literature on this subject is controversial. Although anal sex increases the risk of transmission of infections such as HIV^28^ , a study points out that even though male homosexuals have a higher number of partners, they use condoms more regularly^29^ . Another study found that regular condom use during anal sex is generally less frequent than during vaginal sex, suggesting that the motivation for its use is the prevention of pregnancy, not the prevention of STIs^17^ . These associations can vary greatly from one population to another, as well as depending on the definition of RSB used in the study.

Following a religious doctrine was also not associated with risky sexual behavior, disagreeing with the literature^30^ . Religions, in general, promote sexual abstinence and monogamy and they do not encourage condom use. Moreover, this association may also vary depending on the population, the definition of RSB used, and over time.

The prevalence of sexually transmitted infections at some point in life was 9%, agreeing with another study that evaluated Brazilian undergraduate students from health courses^10^ . The Brazilian Program for the Prevention of STIs was a reference in the world and involved the distribution of condoms, the availability of free pre- and post-exposure prophylaxis, in addition to a series of educational actions aimed at school adolescents. However, STIs has shown signs of resurgence, especially HIV and syphilis^5 , 6^ . The increased survival time of people with HIV in recent decades may have helped reduce the perception of risk and, along with recent cuts in government actions, caused relaxation in prevention.

Among the limitations of the study, we cite that our study covered a wide scope of health issues, being sexual behavior only one of them, which made it impossible to explore different times of recall. Memory bias was minimized by considering a short time, and the anonymity of responses contributed to the veracity of sensitive information. The available studies, in general, address convenience samples or students of health courses; thus, despite the losses, especially in the area of the exact and applied social sciences, our study advanced in the characterization of RSB in undergraduate students.

Standardization of RSB measurement is fundamental for the analysis of consistency of the findings. Some more conservative definitions, which consider a greater diversity of risky behaviors (e.g., having more than 10 sexual partners in life and having had sex after alcohol consumption or some illegal substance, as well as with little or recently known person) or longer recall time, corroborate the perpetuation of taboos and social stigmas regarding the expression of sexuality.

The operationalization of RSB used in our study, limited to condom use in the last sexual intercourse, provides a punctual measure. We considered that the operationalization of the outcome with a focus on health risk was adequate. However, we recommend that future studies further detail the condom use occurrence over a longer period (three months).

Moreover, studies with undergraduate students require logistics capable of avoiding losses, such as a fast field work, which does not exceed one semester and that excludes enrolled students who are not regularly attending university. We believe it is also necessary to expand knowledge about the use of smartphone applications to seek for sex, verifying the relevance of use frequency and the profile of those who use these resources, as well as if the association with RSB is also found in other populations of undergraduate students.

The prevalence of RSB in freshmen undergraduate students was relevant and shows that policies for the institutionalization of sex education in schools are necessary, as well as the updating of concepts and repertoire of factors that can impact sexual behaviors, such as the use of smartphone applications. Moreover, it is important to resume government campaigns to prevent STIs with a focus on young adults, including the availability of condoms in the university environment, aiming at stopping the increase in the rates of preventable infections.
