# Supplementary material for: Risky sexual behavior and associated factors in undergraduate students in a city in Southern Brazil
Source: Rev Saude Publica. 2020 Apr 3;54:41. doi: 10.11606/s1518-8787.2020054001709 (PMC7135094; doi:10.11606/s1518-8787.2020054001709)
Supplement: Translation

# Translation: Comportamento sexual de risco e fatores associados em universitários de uma cidade do Sul do Brasil

**Authors:** Débora Dalmas Gräf, Marilia Arndt Mesenburg, Anaclaudia Gastal Fassa

**Affiliations:** I Brasil Universidade Federal de Pelotas. Programa de Pós-Graduação em Epidemiologia. Pelotas, RS, Brasil; II Brasil Universidade Federal de Ciências da Saúde de Porto Alegre. Porto Alegre, RS, Brasil; III Brasil Universidade Federal de Pelotas. Faculdade de Medicina. Departamento de Medicina Social. Pelotas, RS, Brasil

**Keywords:** Saúde do Estudante, Universidades, Comportamentos de Risco à Saúde, Sexo sem Proteção, Infecções Sexualmente Transmissíveis

## Abstract

**OBJETIVO:**

Descrever o comportamento sexual de ingressantes universitários de acordo com características demográficas, econômicas, psicossociais e comportamentais, e avaliar a prevalência de comportamento sexual de risco e seus fatores associados.

**MÉTODOS:**

Estudo de delineamento transversal, do tipo censo, com universitários maiores de 18 anos, de 80 cursos de graduação da Universidade Federal de Pelotas (UFPel), no RS, que ingressaram no primeiro semestre de 2017 e que permaneceram matriculados no segundo semestre. Avaliou-se o comportamento sexual de risco entre os estudantes que relataram já ter tido relações sexuais alguma vez na vida, considerado quando relatado mais de um parceiro sexual nos últimos três meses e não ter utilizado preservativo na última relação.

**RESULTADOS:**

A prevalência de comportamento sexual de risco foi de 9% (IC95% 7,6–10,5). Estudantes do sexo masculino apresentaram mais comportamento de risco do que estudantes do sexo feminino, com prevalência de 10,8% e 7,5%, respectivamente. Dos universitários, 45% não utilizaram preservativo na última relação e 24% tiveram dois parceiros ou mais nos últimos três meses. Os aplicativos de celular para fins sexuais nos últimos três meses foram utilizados por 23% dos estudantes. O comportamento sexual de risco esteve associado com sexo, idade da primeira relação sexual, frequência de consumo de bebidas alcoólicas, consumo de substâncias psicoativas antes da última relação e uso de aplicativos de celular para fins sexuais.

**CONCLUSÃO:**

Embora se espere que os universitários sejam uma população informada, a prevalência de comportamento sexual de risco foi importante, indicando a necessidade de ampliação do investimento público em ações de educação sexual e conscientização.

## INTRODUÇÃO

Os comportamentos sexuais de risco (CSR), como relação sexual desprotegida e multiplicidade de parceiros, são mais frequentes entre adolescentes e jovens adultos (entre 15 e 24 anos). Fatores ligados ao ingresso no ambiente universitário podem aumentar a ocorrência de CSR, visto que implicam em uma série de modificações sociais na vida do indivíduo^1^ .

Os CSR podem ter como consequência as infecções sexualmente transmissíveis (ISTs)^2 , 3^ e a gravidez não planejada. As ISTs estão entre as condições agudas mais prevalentes no mundo, com cerca de um milhão de casos novos por dia^4^ . O Brasil, nos últimos anos, vem apresentando um recrudescimento das ISTs, especialmente do vírus da imunodeficiência humana (HIV) e da sífilis, com aumento expressivo entre os jovens de 15 a 29 anos^5 , 6^ . As principais consequências das ISTs são infertilidade, gravidez ectópica, natimortos, doença inflamatória pélvica e implicações neurológicas e cardiovasculares em adultos^4^ . Já a gravidez não planejada é particularmente problemática em faixas etárias mais jovens, pois compromete a conclusão da vida escolar e acadêmica, além de aumentar o risco de complicações na gestação^7^ .

No Brasil, a avaliação de uma amostra representativa de estudantes do ensino médio indicou que 32% não utilizaram preservativo nas relações sexuais ocorridas no mês anterior à pesquisa^8^ . Entre os universitários, o não uso de preservativo na última relação variou entre 85,7% e 38,6%^2 , 9^ . A frequência de estudantes que tiveram entre um e três parceiros sexuais no período de três meses foi de 95% no sexo feminino e 89% no sexo masculino^10^ . Nos Estados Unidos, 48% dos universitários utilizaram preservativo na última relação, e a prevalência de comportamento sexual de risco foi de 14%, considerando-se aqueles que reportaram não ter utilizado camisinha na última relação e ter tido mais de um parceiro nos últimos 12 meses^11^ .

Ao examinar características dos comportamentos sexuais, um estudo conduzido em 31 instituições de ensino superior americanas indicou que 44% dos estudantes tiveram mais de um parceiro nos últimos três meses e 16% usaram substâncias psicoativas (álcool ou drogas ilícitas) antes da última relação sexual^12^ . Em universitários brasileiros o uso de substâncias psicoativas antes da última relação foi semelhante, ficando em torno de 15%^2^ . Na cidade de Pelotas, uma pesquisa com adolescentes entre 15 e 18 anos apontou que 10,7% ingeriram bebidas alcoólicas antes da última relação e apenas 56% dos adolescentes usaram preservativo nas últimas três relações sexuais^13^ .

O não uso de preservativos em estudantes, tanto escolares quanto universitários, esteve positivamente associado ao sexo masculino, à ingesta de bebidas alcoólicas e à multiplicidade de parceiros^1 , 14^ e inversamente associado com a idade do indivíduo e com o nível socioeconômico^15 , 16^ .

Os estudos que avaliam comportamento sexual de risco publicados até o momento no Brasil são predominantemente em adolescentes escolares. Os que avaliaram universitários tiveram abordagem descritiva e sua amostra foi, em sua maioria, composta por alunos de cursos da saúde ou então de conveniência, com foco na avaliação de outros desfechos relacionados ao comportamento sexual, como o nível de conhecimento a respeito de ISTs^9 , 16^ . Além disso, o impacto das características psicossociais dos universitários, como a orientação sexual e a identidade de gênero, a variabilidade entre as áreas do conhecimento e o papel da tecnologia sobre os comportamentos sexuais, é pouco abordado.

Este estudo tem por objetivo identificar as principais características do comportamento sexual dos ingressantes universitários de acordo com o sexo e verificar a prevalência de comportamento sexual de risco, bem como os principais fatores sociodemográficos e comportamentais associados, em um censo de ingressantes da Universidade Federal de Pelotas (UFPel), localizada no Sul do Brasil.

## METODOLOGIA

Foi realizado um estudo transversal, do tipo censo, com estudantes ingressantes no ensino superior em cursos presenciais da Universidade Federal de Pelotas (UFPel), ofertados no primeiro semestre letivo de 2017. No total, foram ofertadas 3.424 vagas em 83 cursos de graduação. O censo foi realizado no contexto do consórcio de pesquisa do Programa de Pós-Graduação em Epidemiologia da UFPel, um método de coleta de dados de uma população única para o desenvolvimento do estudo de uma turma de mestrandos.

Foram incluídos nesta pesquisa estudantes que ingressaram na UFPel no primeiro semestre de 2017, estavam regularmente matriculados em um curso de graduação presencial no segundo semestre letivo de 2017 e compreendiam a língua portuguesa. Foram excluídos os estudantes menores de 18 anos e os que tinham aulas ministradas fora da cidade de Pelotas ou Capão do Leão.

A coleta de dados foi realizada em horário de aula, de forma anônima, por meio de um questionário autopreenchido em *tablets* utilizando a ferramenta *Research Electronic Data Capture* (REDCap). O instrumento de pesquisa incluiu questões demográficas, econômicas, psicossociais, do contexto acadêmico e comportamentais, além da descrição do comportamento sexual dos estudantes.

Com base no *Global School-Based Student Health Survey* (GSHS) e no *Youth Risk Behavior Survey* (YRBS), foi elaborado um questionário com dez perguntas objetivas para caracterizar o comportamento sexual, aplicado apenas aos estudantes que já haviam iniciado a vida sexual. Esse questionário avaliou idade da primeira relação sexual, número de parceiros nos últimos três meses, uso de preservativo na última relação, prática de sexo anal na última relação, uso de álcool ou drogas ilícitas na última relação, uso de método contraceptivo, realização do teste de HIV alguma vez na vida, motivo da realização do teste, diagnóstico de IST alguma vez na vida e uso de aplicativos de celular com a finalidade de ter relações sexuais nos últimos três meses.

Considerou-se como comportamento sexual de risco ter mais de um parceiro sexual nos últimos três meses aliado a não utilizar preservativo (camisinha masculina ou feminina) na última relação sexual. Essa operacionalização do desfecho visou restringir a avaliação a aspectos objetivos relacionados ao risco à saúde do indivíduo.

As variáveis de exposição foram sexo biológico (feminino, masculino), idade (categorizada na análise em 18 a 19 anos, 20 a 22 anos, 23 a 25 anos, 26 anos ou mais), cor da pele (branca, preta, parda/outra), orientação sexual (heterossexual/assexual, homossexual, bissexual), identidade de gênero (cisgênero, transgênero, não binário), status de relacionamento (não se relacionando, ficando, namorando, morando com o cônjuge/namorado), classe econômica (classificação da Associação Brasileira das Empresas de Pesquisa – ABEP), tipo de ensino médio cursado (público, privado), onde morou no ano anterior ao ingresso na UFPel (Pelotas, outra cidade do Rio Grande do Sul, outro estado do Brasil/outro país), se segue alguma doutrina religiosa (não, sim), com quem mora (familiares, amigos/colegas, sozinho), consumo de tabaco (nunca fumou, fumante, ex-fumante), frequência de consumo de álcool (nunca, uma vez por mês ou menos, duas a quatro vezes por mês, duas a três vezes por semana, quatro ou mais vezes por semana) e área do conhecimento ao qual o curso do universitário pertence (ciências exatas e da terra/agrárias, ciências da saúde e biológicas, ciências sociais aplicadas e humanas, linguística, letras e artes).

Primeiramente, realizou-se análise descritiva das características demográficas, econômicas, psicossociais, acadêmicas e comportamentais dos estudantes por meio de frequência absoluta e relativa. O teste do qui-quadrado para heterogeneidade foi utilizado para avaliar as diferenças entre os sexos.

Para avaliar fatores associados à ocorrência de comportamento sexual de risco, procedeu-se à análise multivariável, utilizando regressão de Poisson com variância robusta, uma vez que essa é a melhor alternativa para a análise de estudos transversais com desfechos dicotômicos e fornece uma boa estimativa da razão de prevalências^19^ . A análise seguiu o modelo conceitual previamente definido, com seleção para trás. Incluiu-se, no primeiro nível, características demográficas, econômicas e psicossociais; no segundo nível, variáveis do contexto acadêmico e idade da primeira relação sexual e, no terceiro nível, aspectos comportamentais. As variáveis associadas ao desfecho com valor-p < 0,20 foram mantidas no modelo multivariável para controle de fatores de confusão. Foram consideradas significativas as associações com valor-p < 0,05. A análise dos dados foi realizada no programa estatístico *Stata Statistical Software,* versão 14.0.

O presente estudo está de acordo com a resolução vigente nº 466/2012 do Conselho Nacional de Saúde e foi aprovado pelo Comitê de Ética em Pesquisa da Faculdade de Medicina da UFPel, parecer de número 2.352.451.

## RESULTADOS

No primeiro semestre de 2017, 3.212 alunos ingressaram em cursos de graduação da UFPel. Desses, 2.706 permaneciam matriculados na ocasião do início do estudo, o qual se estendeu entre novembro de 2017 e julho de 2018. Ao final do censo de ingressantes universitários, obteve-se 1.865 alunos entrevistados, configurando uma taxa de resposta de 69%. Dos 31% restantes, apenas 1,8% foram recusas. Os demais foram alunos não encontrados durante o período do trabalho de campo após várias tentativas. Aproximadamente 47% das perdas eram do sexo feminino, 24% tinham entre 18 e 19 anos, 29% tinham entre 20 e 22 anos e 46,7% tinham 23 anos ou mais. A área de conhecimento com maior número de perdas foi a de ciências exatas e da terra/agrárias, com 38,3%, seguida das ciências sociais aplicadas e humanas, com 38,4%, linguística, letras e artes, com 16%, e ciências da saúde e biológicas, com 11%. Os altos índices de absenteísmo e de taxas de desistência da universidade do primeiro para o segundo semestre letivos, muitas vezes sem formalização, limitaram a taxa de resposta.

Dentre os respondentes da pesquisa, 1.547 alunos (83,5% dos entrevistados) afirmaram já ter mantido relações sexuais. Desses, 72,2% eram brancos, 37,5% tinham entre 18 e 19 anos, 44,3% eram da classe econômica B, 36,4% estavam namorando e 13,4% eram casados ou dividiam moradia com o cônjuge. A orientação sexual de minorias (homossexual ou bissexual) foi relatada por 22,2%, e 10,4% não se identificaram com o gênero foi atribuído ao nascer (transgênero ou não binário). Quanto à área de conhecimento, 28,3% estavam inseridos em cursos das ciências exatas, da terra e agrárias, 18% das ciências da saúde e biológicas, 35,2% das ciências sociais aplicadas e humanas e 18,5% da área de linguística, letras e artes ( Tabela 1 ). Dos estudantes, 37% tiveram sua primeira relação antes dos 15 anos. Quanto ao uso de substâncias durante a última relação, 15% utilizaram alguma bebida alcóolica, 2,7% alguma droga ilícita e 5,8% ambos. Cerca de 12% dos universitários praticaram sexo anal durante a última relação sexual, e 22% usaram aplicativos de celular com o objetivo principal de ter relações sexuais nos últimos três meses. Segundo os entrevistados, 23% tiveram dois ou mais parceiros sexuais nos últimos três meses, e 45% não utilizaram camisinha na última relação sexual ( Tabela 2 ).

**Tabela 1:** Caracterização dos ingressantes universitários de acordo com o sexo biológico (N = 1.547), Universidade Federal de Pelotas, 2017–2018.

Características	Total	Masculino	Feminino	Valor-p^a^
		
	N (%)	N (%)	N (%)	
Idade				
18 a 19 anos	577 (37,5)	252 (35,6)	325 (39,0)	0,293
20 a 22 anos	509 (33,1)	228 (32,4)	280 (33,6)	
23 a 25 anos	189 (12,3)	95 (13,5)	94 (11,3)	
26 anos ou mais	263 (17,1)	128 (18,2)	134 (16,1)	
Cor da pele				
Branca	1.116 (72,2)	508 (71,8)	606 (72,6)	0,507
Preta	201 (13,0)	88 (12,4)	113 (13,5)	
Parda ou outra	228 (14,8)	112 (15,8)	116 (13,9)	
Status de relacionamento				
Não está se relacionando	509 (33,0)	282 (39,8)	227 (27,3)	<0,001
Ficando	266 (17,2)	130 (18,4)	136 (16,3)	
Namorando	562 (36,4)	212 (29,9)	350 (42,0)	
Morando com cônjuge ou namorado	206 (13,4)	84 (11,9)	120 (14,4)	
Orientação sexual				
Heterossexual ou assexual	1.198 (77,8)	575 (81,2)	622 (74,9)	< 0,001
Homossexual	129 (8,4)	86 (12,2)	43 (5,2)	
Bissexual	212 (13,8)	47 (6,6)	165 (19,9)	
Identidade de gênero				
Cisgênero	1.382 (89,6)	626 (88,4)	756 (90,5)	0,385
Transgênero	110 (7,1)	56 (7,9)	54 (6,5)	
Não binário	51 (3,3)	26 (3,7)	25 (3,0)	
Classe econômica^b^				
A	221 (15,0)	115 (17,1)	106 (13,2)	0,062
B	653 (44,3)	303 (45,1)	349 (43,6)	
C	536 (36,3)	223 (33,2)	312 (39,0)	
D e E	65 (4,4)	31 (4,6)	34 (4,2)	
Tipo de ensino médio				
Público	1.136 (73,5)	510 (72,0)	624 (74,6)	0,248
Privado	410 (26,5)	198 (28,0)	212 (25,4)	
Onde morou antes da UFPel				
Pelotas	704 (45,6)	314 (44,3)	388 (46,5)	0,128
Outra cidade do RS	536 (34,7)	239 (33,7)	297 (35,6)	
Outro estado do Brasil ou outro país^c^	305 (19,7)	156 (22,0)	149 (17,9)	
Com quem mora				
Familiares (pais, irmãos ou cônjuges)	938 (60,8)	411 (58,0)	525 (63,0)	0,092
Amigos ou colegas	414 (26,8)	199 (28,1)	215 (25,8)	
Sozinho	192 (12,4)	99 (14,0)	93 (11,2)	
Religião				
Não	1.086 (70,3)	540 (76,2)	546 (65,4)	< 0,001
Sim	460 (29,7)	169 (23,8)	289 (34,6)	
Consumo de tabaco				
Nunca fumou	1.073 (69,4)	469 (66,2)	603 (72,1)	0,002
Fumante	194 (12,6)	111 (15,7)	83 (9,9)	
Ex-fumante	279 (18,0)	129 (19,2)	150 (17,9)	
Consumo de bebidas alcoólicas^d^				
Nunca	126 (8,6)	62 (9,0)	64 (8,3)	0,055
Uma vez por mês ou menos	388 (26,5)	162 (23,4)	226 (29,2)	
Duas a quatro vezes por mês	585 (39,9)	275 (39,8)	309 (39,9)	
Duas a três vezes por semana	296 (20,2)	152 (22,0)	144 (18,6)	
Quatro ou mais vezes por semana	71 (4,8)	40 (5,8)	31 (4,0)	
Área de conhecimento do curso				
Ciências exatas e da terra/agrárias	437 (28,3)	238 (33,6)	198 (23,7)	< 0,001
Ciências da saúde e biológicas	279 (18,0)	115 (16,2)	164 (19,6)	
Ciências sociais aplicadas e humanas	545 (35,2)	227 (32,0)	318 (38,0)	
Linguística, letras e artes	286 (18,5)	129 (18,2)	156 (18,7)	

^a^Teste do qui-quadrado para heterogeneidade para diferenças entre os sexos

^b^Conforme classificação da Associação Brasileira das Empresas de Pesquisa (ABEP)

^c^O número de alunos que moraram em outro país foi n = 3

^d^O número máximo de *missings* foi n = 81 para esta variável

**Tabela 2:** Descrição do comportamento sexual em ingressantes universitários de acordo com o sexo biológico (N = 1.547), Universidade Federal de Pelotas, 2017–2018.

Desfechos	Total	Masculino	Feminino	Valor-p^a^
		
	N (%)	N (%)	N (%)	
Início da vida sexual^b^				
Não	305 (16,5)	124 (14,9)	181 (17,8)	0,102
Sim	1.547 (83,5)	709 (85,1)	836 (82,2)	
Idade da primeira relação				
13 anos ou menos	82 (5,3)	54 (7,7)	28 (3,4)	< 0,001
14 a 15 anos	483 (31,3)	239 (33,9)	244 (29,3)	
16 a 17 anos	583 (37,8)	264 (37,5)	318 (38,1)	
18 a 19 anos	295 (19,1)	113 (16,0)	181 (21,7)	
20 anos ou mais	98 (6,4)	35 (5,0)	63 (7,6)	
Uso de substâncias^c^				
Não	1.178 (76,4)	522 (73,9)	654 (78,5)	0,011
Bebidas alcoólicas	231 (15,0)	107 (15,2)	124 (14,9)	
Drogas ilícitas	42 (2,7)	21 (3,0)	21 (2,5)	
Bebidas alcoólicas e drogas ilícitas	90 (5,8)	56 (7,9)	34 (4,1)	
Prática de sexo anal^c,d^				
Não	1.354 (87,9)	571 (81,1)	781 (93,7)	< 0,001
Sim	186 (12,1)	133 (18,9)	53 (6,3)	
Uso de método contraceptivo^c^				
Não	574 (37,3)	291 (41,3)	283 (33,9)	< 0,001
Pílula anticoncepcional	728 (47,2)	293 (41,6)	433 (51,9)	
Coito interrompido	62 (4,0)	21 (3,0)	41 (4,9)	
Pílula do dia seguinte	48 (3,1)	21 (3,0)	27 (3,2)	
Anticoncepcional injetável	31 (2,0)	11 (1,6)	20 (2,4)	
Dispositivo intrauterino	15 (1,0)	10 (1,4)	5 (0,6)	
Tabelinha	12 (0,8)	2 (0,3)	10 (1,2)	
Outro	26 (1,7)	15 (2,1)	11 (1,3)	
Não sabe	45 (2,9)	41 (5,8)	4 (0,5)	
Uso de método moderno^c,e^				
Não	719 (46,7)	370 (52,5)	349 (41,9)	< 0,001
Sim	822 (53,3)	335 (47,5)	485 (58,1)	
Uso de aplicativos^f^				
Não	1.193 (77,3)	453 (64,2)	738 (88,4)	< 0,001
Sim	350 (22,7)	253 (35,8)	97 (11,6)	
Número de parceiros^d,f^				
Nenhum	218 (14,2)	105 (14,9)	113 (13,6)	< 0,001
Apenas 1 parceiro	950 (61,7)	382 (54,1)	566 (68,0)	
2 a 3 parceiros	245 (15,9)	130 (18,4)	115 (13,8)	
4 ou mais parceiros	127 (8,3)	89 (12,6)	38 (4,6)	
Camisinha^c^				
Não	700 (45,4)	280 (39,7)	418 (50,1)	< 0,001
Sim	842 (54,6)	426 (60,3)	416 (49,9)	
Comportamento sexual de risco^g^				
Não	1.402 (91,0)	630 (89,2)	770 (92,5)	0,025
2Sim	138 (9,0)	76 (10,8)	62 (7,5)	

^a^Teste do qui-quadrado para heterogeneidade para diferenças entre os sexos

^b^Número de observações: 1.862

^c^Na última relação sexual

^d^O número máximo de *missings* foi n = 20 para estas variáveis

^e^Considerou-se pílula, dispositivo intrauterino, anticoncepcional injetável e pílula do dia seguinte

^f^Nos últimos três meses

^g^Mais de um parceiro nos últimos três meses e não ter utilizado camisinha na última relação sexual

Entre os estudantes do sexo masculino, 41,6% tiveram sua primeira relação sexual antes dos 15 anos, 31% tiveram dois ou mais parceiros sexuais nos últimos três meses e cerca de 40% reportaram não ter utilizado preservativo na última relação. Dos entrevistados, 20% praticaram sexo anal na última relação e 35,8% utilizaram aplicativos com a finalidade de ter relações sexuais nos últimos três meses ( Tabela 2 ).

Entre as estudantes do sexo feminino, 33% tiveram sua primeira relação sexual antes dos 15 anos, 18,4% tiveram dois ou mais parceiros sexuais nos últimos três meses e 50% não usaram preservativo na última relação. Das universitárias, 6% praticaram sexo anal na última relação e 11,6% usaram aplicativos com a finalidade de ter relações sexuais.

A prevalência de comportamento sexual de risco entre os ingressantes universitários foi de 9% (intervalo de confiança de 95% [IC95%] 7,6–10,5), sendo 10,8% entre os estudantes do sexo masculino e 7,5% entre as estudantes do sexo feminino ( Tabela 2 ).

Na análise multivariável, os homens tiveram 48% (Razão de Odds [RO] 1,48; IC95% 1,07–2,07) mais comportamento sexual de risco do que as mulheres. A idade com que os estudantes iniciaram a vida sexual esteve inversamente associada ao CSR. A frequência de consumo de bebidas alcoólicas esteve diretamente associada a comportamento sexual de risco, sendo que aqueles que consumiram álcool quatro ou mais vezes por semana tiveram uma chance cinco vezes maior de ter CSR (RO 5,10; IC95% 1,49–17,6). O uso de drogas ilícitas na última relação sexual e o uso de aplicativos de celular aumentou em mais de 100% a chance de apresentar CSR (RO 2,23; IC95% 1,14–4,39 e RO 2,57; IC95% 1,76–3,74, respectivamente). Idade, orientação sexual, onde o estudante morou antes de ingressar na UFPel, uso de tabaco e prática de sexo anal na última relação não estiveram associados à CSR na análise ajustada ( Tabela 3 ).

**Tabela 3:** Fatores associados ao comportamento sexual de risco em ingressantes universitários (N = 1.381), Universidade Federal de Pelotas, 2017–2018.

Características	Prevalência de CSR	CSR (bruto)			CSR (ajustado)		
		
	%	RP	IC95%	Valor-p^a^	RP	IC95%	Valor-p^a^
Nível 1							

Sexo							
Feminino	7,5	1	-	0,024	1	-	0,020
Masculino	10,8	1,44	1,05–1,99		1,48	1,07–2,07	
Cor da pele							
Branca	8,3	1	-	0,200	1	-	0,253
Preta	9,5	1,15	0,72–1,84		1,14	0,69–1,88	
Parda ou outra	11,4	1,44	0,96–2,16		1,42	0,94–2,15	
Identidade de gênero							
Cisgênero	8,7	1	-	0,021	1	-	0,051
Transgênero	8,2	0,95	0,49–1,81		1,04	0,54–2,00	
Não binário	19,6	2,27	1,27–4,06		2,12	1,16–3,87	
Religião							
Não	9,7	1	-	0,127	1	-	0,119
Sim	7,2	0,75	0,51–1,09		0,73	0,49–1,08	
Classe econômica^b^							
A	11,0	1	-	0,114^c^	1	-	0,091^c^
B	9,4	0,86	0,55–1,34		0,88	0,56–1,38	
C	7,5	0,68	0,42–1,11		0,68	0,42–1,11	
D e E	7,7	0,70	0,28–1,77		0,66	0,26–2,68	

Nível 2							

Com quem mora							
Familiares (pais, irmãos ou cônjuges)	7,5	1	-	0,031	1	-	0,137
Amigos ou colegas	11,9	1,59	1,12–2,24		1,42	0,99–2,05	
Sozinho	9,9	1,32	0,81–2,14		1,34	0,83–2,17	
Área de conhecimento do curso							
Ciências exatas e da terra/agrárias	7,3	1	-	0,355	1	-	0,144
Ciências da saúde e biológicas	9,0	1,23	0,74–2,02		1,18	0,70–1,99	
Ciências sociais aplicadas e humanas	9,0	1,23	0,80–1,89		1,32	0,85–2,03	
Linguística, letras e artes	11,3	1,54	0,96–2,45		1,75	1,08–2,84	
Idade da primeira relação							
13 anos ou menos	17,1	1	-	< 0,001	1	-	0,001
14 a 15 anos	12,2	0,72	0,42–1,22		0,80	0,46–1,39	
16 a 17 anos	7,9	0,46	0,26–0,80		0,51	0,28–0,90	
18 a 19 anos	4,8	0,28	0,14–0,56		0,28	0,13–0,60	
20 anos ou mais	5,1	0,30	0,11–0,79		0,34	0,13–0,90	

Nível 3							

Consumo de bebidas alcoólicas							
Nunca	2,4	1	-	< 0,001^c^	1	-	< 0,001^c^
Uma vez por mês ou menos	5,9	2,49	0,76–8,16		2,30	0,69–7,66	
Duas a quatro vezes por mês	6,9	2,90	0,91–9,23		2,34	0,72–7,65	
Duas a três vezes por semana	17,2	7,23	2,30–22,7		4,30	1,34–13,8	
Quatro ou mais vezes por semana	25,4	10,65	3,25–34,9		5,10	1,49–17,6	
Uso de substâncias na última relação							
Não	6,1	1	-	< 0,001	1		0,024
Bebidas alcoólicas	16,1	2,63	1,81–3,80		1,56	1,06–2,33	
Drogas ilícitas	19,1	3,11	1,60–6,03		2,23	1,14–4,39	
Bebidas alcoólicas e drogas ilícitas	23,3	3,81	2,46–5,89		1,49	0,91–2,47	
Uso de aplicativos nos últimos três meses							
Não	5,6	1	-	< 0,001	1	-	< 0,001
Sim	20,6	3,71	2,71–5,06		2,57	1,76< –3,74	

CSR: comportamento sexual de risco (mais de um parceiro nos últimos três meses e não ter utilizado camisinha na última relação sexual); RP: razão de prevalências

^a^Teste do qui-quadrado para heterogeneidade

^b^Conforme classificação da Associação Brasileira das Empresas de Pesquisa (ABEP)

^c^Teste do qui-quadrado para tendência

Aproximadamente 9% dos universitários tiveram alguma infecção sexualmente transmissível alguma vez na vida. Entre os estudantes que tiveram ISTs, 34,8% referiram HPV, 15,6% herpes genital e 13,3% gonorreia, sendo a herpes mais prevalente no sexo feminino e a gonorreia no sexo masculino ( Tabela 4 ). Entre os universitários, 38% relataram já ter realizado teste de HIV alguma vez na vida. Os motivos mais comuns para a realização do teste foram manter relações sexuais desprotegidas (26%), doação de sangue e solicitação médica (ambos com 15,7%), além de campanhas governamentais (13%).

**Tabela 4:** Frequência de infeções sexualmente transmissíveis e realização de teste de HIV alguma vez na vida em ingressantes universitários, Universidade Federal de Pelotas, 2017–2018.

Desfechos	Total	Masculino	Feminino	Valor-p^a^
		
	N (%)	N (%)	N (%)	
Infecção sexualmente transmissível (N = 1.540)				
Não	1.405 (91,2)	643 (91,3)	761 (91,2)	1,000
Sim	135 (8,8)	61 (8,7)	73 (8,8)	
Infecção sexualmente transmissível (N = 135)				
HPV	47 (34,8)	18 (29,5)	28 (38,4)	0,004
Herpes genital	21 (15,6)	6 (9,8)	15 (20,6)	
Gonorreia	18 (13,3)	14 (23,0)	4 (5,5)	
Clamídia	7 (5,2)	3 (4,9)	4 (5,5)	
Tricomaníase	7 (5,2)	1 (1,6)	6 (8,2)	
Sífilis	6 (4,4)	4 (6,6)	2 (2,7)	
HIV/AIDS	5 (3,7)	5 (8,2)	-	
Outra	24 (17,8)	10 (1,4)	14 (1,7)	
Realização do teste de HIV (N = 1.542)^b^				
Não	961 (62,3)	444 (63,0)	517 (61,9)	0,673
Sim	581 (37,7)	261 (37,0)	318 (38,1)	
Principal motivo para a realização do teste de HIV (N = 580)				
Relação sexual desprotegida	152 (26,2)	82 (31,4)	70 (22,1)	< 0,001
Doação de sangue	91 (15,7)	48 (18,4)	43 (13,6)	
Solicitação médica	91 (15,7)	29 (11,1)	62 (19,6)	
Campanhas governamentais	76 (13,1)	37 (14,2)	39 (12,3)	
Pré-natal	43 (7,4)	1 (0,4)	42 (13,2)	
Solicitação do parceiro	13 (2,2)	9 (3,5)	4 (1,2)	
Exposição ocupacional	11 (1,9)	3 (1,1)	7 (2,2)	
Outro	103 (17,8)	52 (19,9)	50 (15,8)	

^a^Teste do qui-quadrado para heterogeneidade para diferenças entre os sexos

^b^O número máximo de *missings* foi n = 20 para esta variável; teste realizado alguma vez na vida

## DISCUSSÃO

Comportamento sexual de risco tem diferentes definições na literatura, dificultando comparações. Além disso, alguns fatores tratados como características do comportamento sexual são abordados em outros estudos como parte do comportamento sexual de risco. O estudo que mais se aproximou da definição deste trabalho foi conduzido em universitários americanos e considerou os mesmos critérios para compor o desfecho, porém com prazo de recordatório para parceiros múltiplos ampliado para 12 meses. A prevalência de CSR encontrada nele foi de 14% e o não uso de preservativo na última relação foi de 52%^11^ , sendo similar aos achados nos ingressantes universitários da UFPel. Essas prevalências em universitários, uma população com nível de informação elevado, reforçam a ideia de que ter informação é um fator necessário, porém não suficiente para modificar comportamentos^18^ .

O estudo é consistente com a literatura que aponta que metade dos universitários fez uso de preservativo^20 , 21^ e 15% ingeriram bebidas alcoólicas na sua última relação sexual^2 , 12^ . Entretanto, um estudo mostrou que mais de 40% dos calouros universitários americanos tiveram parceiros sexuais múltiplos nos últimos três meses, o dobro do encontrado neste trabalho^12^ . Não foram encontrados outros estudos sobre o uso de aplicativos com a finalidade de ter relações sexuais; entretanto, esses recursos estão disponíveis há um período curto de tempo e, provavelmente, seu uso encontra-se em ascensão entre os universitários. Nesse contexto, considerou-se a prevalência elevada.

O comportamento sexual de risco esteve positivamente associado com o sexo masculino, uso de substâncias psicoativas antes da última relação sexual e uso de aplicativos de celular com a finalidade de ter relações sexuais nos últimos três meses. Ele esteve diretamente associado com a frequência de consumo de bebidas alcoólicas e inversamente associado com a idade de início das relações sexuais.

A maior frequência de comportamento sexual de risco no sexo masculino é consistente com a literatura que aponta essa associação independentemente da forma como CSR é avaliado, bem como quando se examinam os componentes do CSR separadamente (menor uso de preservativo e maior número de parceiros)^1 , 8 , 11^ . Ser do sexo masculino implica estar sujeito a um repertório de cobranças e pressões sociais, tais como encorajamento da expressão da sexualidade em nome da “masculinidade” e provação da virilidade e da heterossexualidade, refletindo no aumento do número de parceiros^1 , 22^ . Além disso, alguns homens relatam que o preservativo reduz a sensibilidade durante o ato sexual, contribuindo para o não uso.

Assim como este estudo, a literatura aponta associação inversa entre a idade da primeira relação sexual e o comportamento sexual de risco. Há indícios de que não residir com mãe ou pai aumenta o risco de ter uma sexarca mais cedo. A menor presença e supervisão dos pais pode ter impacto negativo na educação sexual dos filhos^22 , 23^ .

A associação positiva entre consumo de bebidas alcoólicas e uso de substâncias psicoativas com CSR está de acordo com a literatura, a qual aponta que essas substâncias potencializam comportamentos de risco^2 , 10 , 12 , 24^ . Isso ocorre porque substâncias entorpecentes, especialmente o álcool, deprimem o sistema nervoso central, prejudicando as habilidades psicomotoras e o processamento de informações, afetando a percepção do perigo e a capacidade de tomar decisões adequadas^25^ .

A literatura sobre o uso de aplicativos de celular para fins sexuais é escassa, pois esse fenômeno surgiu há aproximadamente 10 anos, com a popularização das redes sociais, e migrou de plataformas abrangentes para comunidades específicas para esse fim. Estudos apontam que, ao facilitar interações sociais entre indivíduos desconhecidos, os aplicativos ampliam a possibilidade de relações entre parceiros eventuais e aumentam a chance de ter relação sexual desprotegida^26 , 27^ . A associação entre uso de aplicativos e CSR chama atenção porque, apesar de esta população ter alta escolaridade e parceiros sexuais eventuais, não faz uso consistente de camisinha.

Todas as características associadas ao CSR – sexo masculino, idade da primeira relação sexual, frequência elevada de consumo de bebidas alcoólicas, uso de substâncias psicoativas antes da última relação e uso de aplicativos de celular com a finalidade de ter relações sexuais – são potenciais marcadores de indivíduos com perfis tomadores de risco^11^ .

A orientação sexual, identidade de gênero e prática de sexo anal não estiveram associadas com o comportamento sexual de risco. A literatura sobre essa temática é controversa. Apesar de o sexo anal aumentar o risco de transmissão de infecções como HIV^28^ , um estudo aponta que homossexuais do sexo masculino têm maior número de parceiros, no entanto fazem mais uso de camisinha^29^ . Outro estudo verificou que o uso de preservativo durante o sexo anal é, em geral, menos consistente do que durante o sexo vaginal, sugerindo que a motivação do uso é a prevenção da gravidez, não a prevenção das ISTs^17^ . Essas associações podem variar bastante de uma população para outra, bem como em função da definição de CSR utilizada.

Seguir uma doutrina religiosa também não esteve associado com o comportamento sexual de risco, contrariando a literatura^30^ . As religiões, em geral, promovem abstinência sexual e monogamia, porém não incentivam o uso de preservativo. Além disso, essa associação também pode variar em função da população, da definição de CSR utilizada e ao longo do tempo.

A prevalência de infecções sexualmente transmissíveis alguma vez na vida foi de 9%, número que está alinhado com outro estudo que avaliou universitários brasileiros de cursos da saúde^10^ . O programa brasileiro de prevenção de ISTs foi referência no mundo e envolveu a distribuição de preservativos, a disponibilização de profilaxias pré e pós-exposição gratuitas, além de uma série de ações educacionais voltadas aos adolescentes escolares. No entanto, as ISTs vêm apresentando indícios de recrudescimento, especialmente o HIV e a sífilis^5 , 6^ . O aumento da sobrevida dos portadores de HIV nas últimas décadas pode ter colaborado para reduzir a impressão do risco e, juntamente com cortes recentes nas ações governamentais, provocado relaxamento na prevenção.

Dentre as limitações do estudo, é importante mencionar que a pesquisa abrangeu um amplo escopo de temas em saúde, sendo o comportamento sexual apenas um deles, o que impossibilitou a exploração de diferentes tempos de recordatório. O viés de memória foi minimizado ao manter-se esse tempo curto, e o anonimato das respostas contribuiu para a veracidade das informações de caráter sensível. Os estudos disponíveis, em geral, abordam amostras de conveniência ou cursos da área da saúde; assim, apesar das perdas, especialmente na área das ciências exatas e sociais aplicadas, este estudo avançou na caracterização do CSR em universitários.

A padronização da medida de CSR é fundamental para a análise de consistência dos achados. Algumas definições mais conservadoras, que consideram uma diversidade maior de comportamentos de risco (por exemplo, ter mais de 10 parceiros sexuais ao longo da vida e ter mantido relações sexuais após o consumo de álcool ou alguma substância ilegal, assim como com pessoa pouco ou recentemente conhecida) ou um tempo de recordatório mais longo, colaboram com a perpetuação de tabus e estigmas sociais a respeito da expressão da sexualidade.

A operacionalização de CSR utilizada neste estudo, limitada ao uso de preservativo na última relação sexual, proporciona uma medida pontual. Considera-se que a operacionalização do desfecho com foco no risco à saúde foi adequada. Entretanto, recomenda-se que futuros estudos acrescentem um detalhamento do uso de preservativo em um período mais longo (três meses).

Além disso, estudos com universitários requerem uma logística capaz de evitar as perdas, como um trabalho de campo rápido, que não extrapole o semestre e que exclua alunos matriculados que não estão efetivamente frequentando a universidade. É preciso também ampliar os conhecimentos sobre o uso de aplicativos com o objetivo de ter relações sexuais, verificando a relevância da frequência do uso e o perfil de quem utiliza esses recursos, bem como se a associação com CSR se mantém em outras populações de universitários.

A prevalência de CSR em universitários foi relevante e demonstra que políticas de institucionalização da educação sexual nas escolas são necessárias, assim como a atualização de conceitos e do repertório de fatores que podem impactar nos comportamentos sexuais, como o uso de aplicativos. Além disso, é importante retomar as campanhas governamentais de prevenção das ISTs com foco em adultos jovens, inclusive com disponibilização de preservativos no ambiente universitário, com o objetivo de frear o aumento das taxas de infeções preveníveis.
